# How Metal Substitution Affects the Enzymatic Activity of Catechol-O-Methyltransferase

**DOI:** 10.1371/journal.pone.0047172

**Published:** 2012-10-08

**Authors:** Manuel Sparta, Anastassia N. Alexandrova

**Affiliations:** Department of Chemistry and Biochemistry, University of California Los Angeles, Los Angeles, California, United States of America; Medical School of Hannover, United States of America

## Abstract

Catechol-O-methyltransferase (COMT) degrades catecholamines, such as dopamine and epinephrine, by methylating them in the presence of a divalent metal cation (usually Mg(II)), and S-adenosyl-L-methionine. The enzymatic activity of COMT is known to be vitally dependent on the nature of the bound metal: replacement of Mg(II) with Ca(II) leads to a complete deactivation of COMT; Fe(II) is slightly less than potent Mg(II), and Fe(III) is again an inhibitor. Considering the fairly modest role that the metal plays in the catalyzed reaction, this dependence is puzzling, and to date remains an enigma. Using a quantum mechanical / molecular mechanical dynamics method for extensive sampling of protein structure, and first principle quantum mechanical calculations for the subsequent mechanistic study, we explicate the effect of metal substitution on the rate determining step in the catalytic cycle of COMT, the methyl transfer. In full accord with experimental data, Mg(II) bound to COMT is the most potent of the studied cations and it is closely followed by Fe(II), whereas Fe(III) is unable to promote catalysis. In the case of Ca(II), a repacking of the protein binding site is observed, leading to a significant increase in the activation barrier and higher energy of reaction. Importantly, the origin of the effect of metal substitution is different for different metals: for Fe(III) it is the electronic effect, whereas in the case of Ca(II) it is instead the effect of suboptimal protein structure.

## Introduction

Catechol-O-methyltransferase (COMT, EC 2.1.1.6) is an enzyme involved in the biology of pain, where its inhibition leads to greater pain sensitivity. The metabolic role of COMT is the inactivation of neurotransmitters and neuroactive xenobiotics possessing a catechol motif, and regulating their amount in the brain and other organs [1], [2]. Proper regulation of catecholamines is critical for the organism survival and wellbeing. Malfunctioning of COMT and improper levels of those neurotransmitters are associated with a number of medical conditions, such as Parkinson disease, anxiety, substance abuse, and schizophrenia. Hence, COMT is a common target for drugs that alter its activity and regulate the level of catecholamines in the body.

The enzyme promotes the transfer of the methyl group from the cofactor, S-adenosyl-L-methionine (SAM), to one of the hydroxyl groups of catechol, or substituted catechol, in the presence of a divalent metal cation (Scheme S1) [3]. The cation naturally employed in COMT is Mg(II). The role of the cation is to merely hold the substrate catechol in a reactive orientation with respect to SAM, suitable for the methylation [4], [5]. Mg(II) can be substituted with Co(II), Mn(II), Zn(II), Cd(II), Fe(II), Fe(III), Ni(II), and Sn(II), [3] but metal replacement sometimes leads to unexplainable changes in the enzyme activity. For example, a seemingly harmless replacement of Mg(II) with Ca(II) leads to complete deactivation of the enzyme [6]. Also, Fe(III) is an inhibitor of COMT, whereas Fe(II) is only a marginally poorer catalyst than the native Mg(II) [6]. Co(II) and Mn(II), on the other hand, are most effective in catalysis [3]. It is not fully understood whether the change in activity upon metal substitution is due to the catalytic process itself, or other factors. For example, Mg(II) itself becomes inhibitory for COMT at increased concentrations. The inhibition mode is reported to be mixed or noncompetitive, [1], [2] which implies that Mg(II) can not only bind in the catalytically active complex, but also in a different, inhibitory manner. It could well be that the lack of activity in the presence of Ca(II), for example, results from these secondary effects that are more pronounced with Ca(II). The whole picture is complicated and poorly understood. In this study, we focus on just the catalytic process, with the purpose of determining the extent to which the metal-dependent behavior of COMT could be explained by the relative facility of the catalytic process itself.

From a mechanistic point of view, the catalytic cycle of COMT is well understood. Lotta et al. [4] performed a kinetic study of the methylation reaction of dopamine, (−)-noradrenaline, L-dopa, and 3,4-dihydroxybenzoic acid catalyzed by SAM. They showed that, in order for all components to bind in a reactive orientation and for the reaction to occur, the reaction needs to proceed through an ordered sequential mechanism: In the first step, SAM binds the protein and forms a stable complex. Tsao et al. [7] also showed that in the absence of metal and substrate SAM adopts a different docking pose in comparison to the holoenzyme. In the second step, the protein binds a divalent metal cation through coordination to several acidic residues, and without direct interaction between the metal and SAM. In the last step, the catechol-containing substrate coordinates to the metal and establishes a network of hydrogen bonds with the residues at the active site [4]. In this conformation of the complex, the methyl group attached to the S atom in SAM points toward the hydroxyl group of the substrate, ready for transfer. This arrangement was confirmed when the crystal structures of rat (1994) and human (2008) COMT bound with SAM, Mg(II) and a substrate analog, 3,5-dinitrocatechol, were crystallized [5]. By using isotopic labeling of the methyl group (deuterium and carbon-13), Hegazi et al. were able to demonstrate that the methyl transfer is the rate-determining step in catalysis, with an S_N_2-like transition state (TS) in which the methyl group is located symmetrically and “tightly” between the leaving group and nucleophile [8].

Several theoretical studies confirmed these experimental findings and contributed to our understanding of the mechanism. In 1997, ab initio and semiempirical molecular orbital theory methods were used to examine the methylation of catechol by SAM in various media. It was concluded that, in the gas phase, the reaction is facile, whereas in solution, it is very slow, with an estimated reaction barrier of 37.4 kcal/mol [9]. The authors suggested that the ability of COMT to catalyze the reaction is due to the desolvation and optimal arrangement of the reactants [9]. Kollman and coworkers combined quantum mechanical (QM) and free energy calculations to investigate the catalyzed methyl transfer in the Mg(II) form of COMT and obtained the activation energy of 24.5 kcal/mol [10], [11]. They also confirmed that, in solution, the activation energy increases by 14–18 kcal/mol [10], [11]. Roca et al. used both molecular dynamics (MD) and QM simulations to investigate the enzymatic mechanism and also identified TS and reaction energetics that were in a good agreement with the experiment [12]–[15].

Several aspects of protein structure and dynamics were found to play a role in catalysis. Via MD simulations, it was shown that the formation of the near attack conformations that are structurally close to the TS of the reaction in COMT was correlated with the motion of the protein [16], [17]. Besides the mechanistic implication, this study pointed at the importance of taking into account the large scale protein dynamics in computational studies of COMT. Further, bringing the ground state of the system closer to the TS was predicted to be assisted by Met40, Tyr68, and Asp141 near the active site, although these residues do not provide the actual TS stabilization, according to QM calculations on the cluster model of the active site [18]. Of an additional importance is the residue Lys144 in its uncharged form, whose role was suggested to deprotonate the catecholic substrate when it binds to the active site [19].

To the best of our knowledge, all the attempts to model catalysis in COMT have focused on the native Mg(II) dependent enzyme, whereas the impact of the metal substitution on the catalytic activity of COMT has not been investigated. In view of the inhibiting properties of some of the metals that can bind to COMT, and the important biological implication of COMT inhibition, it is critical to understand why some metals promote catalysis and some deprive it. To date, no understanding of this kind has emerged. In this work, we aim at explicating the effect of metal substitution on the rate-determining step of catalytic methyl transfer in COMT. We study four representative metals: catalytic Mg(II) and Fe(II), and inhibiting Ca(II) and Fe(III).

## Theoretical Methods

In this study, we employ our recently developed QM/DMD method [20], DMD standing for discrete molecular dynamics [21]–[24]. DMD is an exceptionally successful method for sampling biological molecules and their complexes. It is classical force field based. No explicit solvent is used in DMD, but solvation is included in the averaged way into the force field in use. We have previously demonstrated that QM/DMD is capable of recapitulating the native states of metalloproteins, recover the native state if provided with a distorted starting structure, predict metalloprotein structure upon amino acid mutations in, or near the active site, and upon metal replacement [20]. Specifically, we used the Fe(II)/Fe(III) protein, rubredoxin, for extensive testing of the method. QM/DMD was capable of quickly stabilizing its structure in the basin very close to the X-Ray structure, including fine structural details, such as lengths of weak hydrogen bonds. The produced ensemble was shown to be a good starting point for more intricate calculations of electronic effects, such as changes in reduction potential of Fe upon mutations in its second coordination sphere. We also distorted the protein by removing Fe, protonating the four Cys residues coordinating it, and relaxing the structure with pure DMD. To the resultant structure, we then added Fe and deprotonated the four Cys again. QM/DMD returned the protein into its native state, in terms of backbone and active site RMSDs, and QM and DMD energies, by approximately 5^th^ iteration. A key feature of QM/DMD is an affordable extensive sampling of the protein on the time-scales of several nanoseconds, achieved via DMD, in combination with accurate ab initio description of the active site [20]. QM/DMD is a variant of QM/MM, [21]–[24] an established general approach to studies on metallo-enzymes. However, affordability makes it attractive as compared to other often equally capable QM/MM methods. We use QM/DMD to find the set of representative structures occurring in the optimal equilibrated ensembles, characteristic of different metal variants of COMT.

### The QM/DMD approach to partitioning the protein

**Figure 1:**
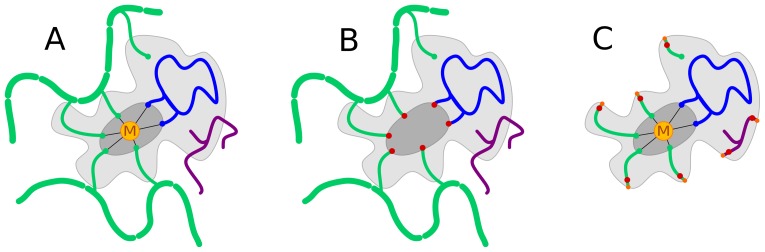
Schematic representation of the DMD/QM domains. A) The dark shaded area defines the *QM-only* domain, the light shaded area is the *QM-DMD* domain, and the rest of the system constituted the *DMD-only* domain. Protein backbone is shown as thick green lines. Amino acids holding the metal (**M**) are depicted as thin green lines. The catechol substrate coordinated to **M** is shown in blue. SAM is shown in purple; notice that only part of it is included in the *QM-DMD* domain. B) Representation of the system as seen during the DMD phases of the simulation: red dots depict atoms that are frozen. C) Schematic model of the system during QM calculations: atoms that are bordering with the *DMD-only* domain are frozen and their valences are saturated with hydrogen atoms (orange dots).

The coupling between QM and DMD in the QM/DMD methods is achieved via partitioning the whole system into three domains, and then using the “breathing” QM-DMD boundary that circumvents parts of the protein significantly different in size, depending on the stage of the simulation. Figure 1A shows how the protein is divided into three domains, in a generic way. The metal cation, **M**, and the portion of the active site immediately surrounding it constitute *QM-only* domain (the dark grey region in Figure 1), which moves only during the QM phase of the simulation (see below), and is not allowed to be moved by classical DMD. This constraint eliminates the dependence of the method on any parameterization of the classical force field for the metal and makes the method fully transferrable. The light shaded area is the shared *QM-DMD* domain, which can be moved by both QM and DMD, depending on the stage of the run. The *QM-DMD* domain is a minimalistic and chemically meaningful cluster model of the active site. It includes the amino acids holding the metal, the substrate, a portion of the SAM moiety, and a few H-bond donors and acceptor side chains important for the retention of the proper structure of the active site in QM calculations, as described below. The rest of the system, including most of the protein macromolecule and a large portion of SAM, constitutes the *DMD-only* domain.

Simulations are performed in an iterative fashion, where the DMD and QM machineries alternate, as described in detail below. Figure 1B shows the representation of the system during the DMD simulation: the *QM-only* region is fixed and formally unseen by DMD, but the protein and substrate can move and adjust to the position of the fixed “metal + bonded atoms” moiety. Figure 1C shows a schematic model of the system during QM calculations: important parts of the active site are extracted from the protein. Atoms that are bordering with the *DMD-only* region are frozen in the positions dictated by the rest of the protein and their valences are saturated with hydrogen atoms. The existence of the large domain shared by DMD and QM, together with an iterative swapping between the QM and DMD models, ensure the adaptive coupling between the protein and the active site.

### System preparation

The initial structure of COMT was obtained from the protein data bank (PDB code: 3BWM, 1.98 Å resolution) [5a]. In this structure, an inhibitor, 3,5-dinitrocatechol, is coordinated the Mg(II) cation. The inhibitor is very close in structure to the substrate of COMT. Therefore, its binding pose serves as an appropriate starting point for studying the catalytic process.

**Figure 2:**
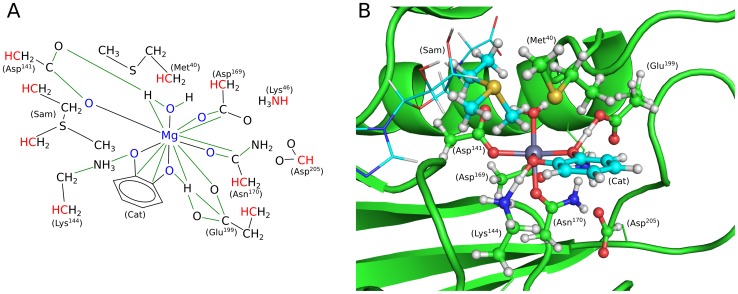
QM/DMD setup for COMT. A) Topological representation of the system as treated during the QM calculations (*QM-DMD* domain). The color red marks the atoms whose positions are frozen during QM calculations, to retain the structure imposed by the rest of the protein. With the blue color, the atoms in the *QM-only* domain are marked. Green lines show additional constrains that are imposed during the DMD simulation, to retain the chemistry determined at the QM level of theory. B) *QM-only* domain embedded in the protein.

There is a network of bonding and nonbonding interactions in the active site that need to be accounted for in QM calculations. The metal is surrounded by the side chains of Asp141, Asp169, Asn170; a water molecule and the substrate complete the octahedral coordination. The side chains of Lys144 and Glu199 interact directly with the substrate by means of H-bonds. The methyl group from SAM points toward the substrate. The side chains of Lys46 and Asp205 interact with Asp169 and Asn170. Finally, the side chains of Met40 shields the active site from solvent interactions. Hence, the region included in the QM calculation (the *QM-DMD* domain) contains the metal center, the side chains, or parts of side chains of Met40, Lys46, Asp141, Asp169, Asn170, Lys144, Glu199, and Asp205, a water molecule, a portion of the cofactor SAM (CH_3_S(CH_3_)CH_2_CH_3_), and cathecol (Figure 2). The truncation of the aforementioned amino acids in the QM calculations occurred at the C_ α_-C_α_ bond, except for Lys46 in which the C_ε_-N_ζ_ bond was cut, Lys144 (C_γ_-C_δ_), and Asp205 (C_β_-C_γ_). Whenever a bond was truncated, the atom included in the QM region was saturated with a hydrogen atom positioned along the original bond at a distance equal to 0.7052*R(C-C/N). All saturating hydrogens and their bond partners were frozen during the QM optimization of the active site to retain the geometry imposed by the rest of the protein (Figure 2).

For the description of the *QM-DMD* domain during the DMD phase, the standard DMD parameterization for all-atom systems was used for the most part [25]–[28]. However, a set of additional constrains, as derived from the QM calculations, were imposed to direct the sampling procedure. In particular, for each pair of atoms connected by green lines in Figure 2A, the sampling of the interatomic distance was limited to the interval defined by the QM optimized value ±0.01Å. Furthermore, considering that this study does not focus on the docking of SAM, we froze most of its non-hydrogen atoms during the DMD simulations, to preserve the configuration as it was found in the crystal structure. In view of the weak binding of SAM to the protein, this constraint was found necessary, in order to not loose SAM to the solution during our long DMD simulations.

### Simulation details

Each simulation started from a short DMD run of 1,000 DMD time unit (t.u.) (1 t.u. roughly corresponds to 50 fs), at the temperature of T = 0.10 kcal/(mol?K), with a high heat exchange rate of the protein with the bath for 10 t.u.^−1^, using Andersen thermostat [29]. This was found to be an efficient method to remove clashes from the structure found in the pdb starting structure [30]. Afterwards, the QM/DMD simulation on the prepared protein was initiated by the DMD phase. The temperature was ramped to 0.20 kcal/(mol?K), and then decreased stepwise (5 steps, consisting of 500 t.u. each) to anneal and equilibrate the structure. The data collection was done during the subsequent 10,000 t.u. performed at T = 0.10 kcal/(mol?K), with the heat exchange rate set at 0.1 t.u.^−1^. The snapshots of the system were saved every 10 DMD t.u., yielding a total of 1,000 poses of the system per DMD phase. The DMD ensemble was clustered into 5 clusters, according to geometric similarity, based on the Kabsch [31] RMSD for all pairwise snapshot structures and by applying a hierarchical clustering algorithm [32]. For each cluster, both the structure closest to the centroid and the one with the lowest DMD energy were used as representatives. The overall QM/DMD simulation for each protein corresponded to approximately 10.5 ns of dynamics.

For each structure, the QM-DMD domain was extracted, capped, and its single point energy was calculated at the QM level. For the QM calculations we used Density Functional Theory (DFT), in the specific the BP86 [33]–[37] formulation of the exchange and correlation functional. A double ζ quality basis set (def2-SV(P)) [38] for H, C, N, O, S, and a triple ζ quality basis set (def2-TZVPP) [39] for the metals were used. Resolution of Identity (RI), [40] and Multipole Accelerated Resolution of Identity (MARI-J) [41] as implemented in *Turbomole* [42] were exploited to speed up the calculations. Empirical dispersion correction for DFT calculations were included both in the energy and gradient evaluations [43]. Based on the combined scoring index that reflected the QM and DMD energies characteristic of every structure, [20] a single structure was chosen and partially optimized at the QM level. The atoms bridging the *QM-DMD* region with the rest of the protein were fixed during the optimization.

The optimized *QM-DMD* domain was then uncapped and reinstalled in the protein. The QM-DMD boundary shrank back to go around the *QM-onl*y region. The simulation continued with the next DMD phase, where the changes in the *QM-DMD* domain had a chance to impact the structure of the rest of the protein. A total of 20 iterations for QM/DMD was found to be sufficient to equilibrate every protein in this study (see Figures S1, S2, S3, S4, S5). In order to improve the convergence of QM/DMD, we implemented a criterion to determine whether the QM output structure of the *n* ^th^ iteration should be used as input for DMD in the (*n*+1)^th^ iteration, or if instead the DMD simulation should start again from the *n* ^th^ input, as follows. At the end of each iteration, the current input and output structures were compared, and the one with the lowest QM energy (computed on a subset of the active site: Lys46, Asp141, Asp169, Asn170, Lys144, Glu199, the metal, substrate and water) was used as input in the subsequent iteration. Even though not all structures were chosen as starting points for the next iterations, they were saved and used for analysis.

The set of structures generated by QM/DMD at the stage of convergence, are then used for the mechanistic investigation. Strictly speaking, these QM/DMD snapshots do not form a canonical ensemble. First of all, the precise energies of all structures are not accessible, and instead structures are characterized by separate QM and DMD energies. Additionally, entropies could be evaluated only approximately, from the clustering algorithm in DMD, but DMD does not include the metal with its immediate coordination. Therefore, both enthalpy and entropy are approximate. The use of snapshots in mechanistic study results in additional uncertainty of the obtained results. However, considering the spread in the calculated values of activation barrier is small (see below), we are quite confident in the quality of our obtained sets of structures.

### QM calculations of reaction energetics

The equilibrated QM/DMD ensembles of structures were used for the mechanistic investigation. TSs and products were identified for each starting structure, using the same level of QM theory as in the QM/DMD simulations. The nature of each stationary point was confirmed with a frequency calculation: no imaginary frequencies confirmed the local minima, and one imaginary frequency with the normal mode going along the reaction coordinate confirmed TSs. All QM calculations were executed using the *Turbomole* package [42]. For all the stationary points, single point calculations were carried at M06^37^ [44]/def2-TZVPP, with the active site embedded into the charge distribution generated by the rest of the protein as implemented in NWChem [45]. The empirical dispersion correction is also included in the QM calculations [43]. Reported reaction energies are ZPE-corrected and averaged over the ensemble. In addition to the aforementioned method, we computed single point energy evaluation for all the stationary points using B3LYP, [46] and TPSSh [47]. The obtained ZPE-corrected energies are then used to distinguish the studied COMT variants. Strictly speaking, it is free energies that have to be correlated with the experimentally observed rates. However, calculations of entropies on the constrained complexes extracted from the entire protein structure are unphysical. Therefore, we make an approximation that just energies would give us a qualitatively correct picture.

## Results and Discussion

### Structural characterization of the COMT reactive complexes

**Figure 3:**
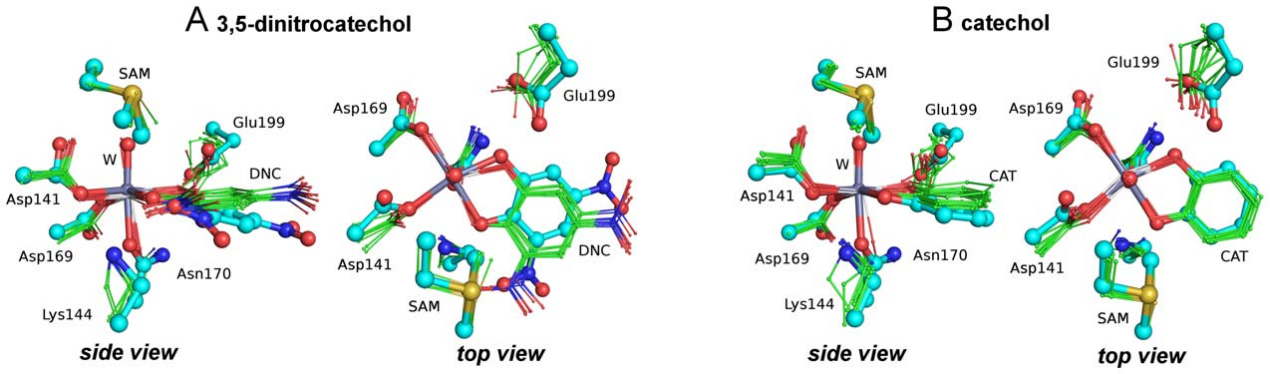
Comparison between the QM/DMD ensembles of active site structures (thin sticks, small spheres), and the X-ray structure (bold sticks, large spheres) for A) 3,5-dinitrocatechol, and B) catechol bound to the Mg(II) form of COMT. Hydrogen atoms, Met40, Lys46 and Asp205 are omitted for clarity.

In order to test the accuracy of the QM/DMD method for modeling of COMT and its derivatives, we first simulated the native Mg(II) form of the enzyme with the bound 3,5-dinitrocatechol inhibitor and SAM. Our goal was to reliably recapitulate the X-ray structure of the protein. Figure 3A shows a portion of the active site structures obtained with QM/DMD (Met40, Lys46 and Asp205, and all the hydrogen atoms have been omitted for clarity) in comparison with the X-ray structure. A good structural agreement is apparent, and, in particular, the side chains interacting with Mg and with the inhibitor are well clustered and closely resemble the X-ray structure. A slight discrepancy is found for the position of the inhibitor: the simulated structures are slightly tilted (as seen from the side view), and rotated (as seen from the top view), as compared with the experimental structure. This results in a tighter interaction between the 3,5-dinitrocatechol and the metal.

On the other hand, when the catechol substrate is bound to Mg, it assumes a conformation that resembles the X-ray structure more closely (see Figure 3B). This suggests that the mentioned subtle rotation of the simulated inhibitor with respect to the experimental structure may be due to the gas-phase approximation ultimately used in the QM phases of QM/DMD, or the basis set superposition error, that induce overestimated interactions between the Lys144 and the nitro group of the 3,5-dinitrocatechol.

Averaged significant structural parameters characterizing the coordination environment of Mg in COMT with bound 3,5-dinitrocatechol or catechol are shown in Table 1, and theoretical predictions are compared to the experiment. Except for the Mg – O(Asp169) bond length, all the simulated values are found within ca. 0.2 Å from the experimental values, confirming good structural agreement. Structural parameters as a function of the bound metal are also collected in Table 1, and the associated simulations are discussed next.

**Table 1:** Averaged structural parameters^a^ characteristic of the active site of COMT as function of the bound metal cation and the substrate: 3,5-dinitrocatechol (DNC), or catechol (Cat). Experimental values for Mg-COMT/3,5-dinitrocatechol are shown for comparison.

	Exper.^b^	Simulated				
Metal	Mg(II)	Mg(II)	Mg(II)	Ca(II)	Fe(II)	Fe(III)
Substrate	DNC	DNC	Cat	Cat	Cat	Cat
Metal – O1(Asp141)	2.13	2.31 (0.37)^c^	2.23 (0.08)	2.51 (0.03)	2.08 (0.05)	2.15 (0.06)
Metal – O2(Asp141)	3.28	3.44 (0.19)^d^	3.43 (0.04)	2.46 (0.05)	3.25 (0.03)	3.27 (0.05)
Metal – O(Asp169)	2.35	1.99 (0.02)	1.99 (0.01)	2.24 (0.04)	2.00 (0.04)	1.94 (0.04)
Metal – O1(Asn170)	2.24	2.12 (0.03)	2.14 (0.03)	2.63 (0.11)	1.96 (0.02)	2.02 (0.07)
						
Metal – O(water)	2.04	2.10 (0.01)	2.11 (0.01)	2.41 (0.01)	2.07 (0.03)	2.05 (0.05)
Metal – O1(Cat)	2.14	2.11 (0.02)	2.08 (0.02)	2.36 (0.02)	1.97 (0.02)	1.92 (0.02)
Metal – O2(Cat)	2.21	2.10 (0.03)	2.14 (0.03)	2.41 (0.02)	2.06 (0.03)	1.98 (0.03)
O2(Cat) – C(SAM)	2.71	2.82 (0.10)	2.59 (0.05)	2.85 (0.06)	2.75 (0.09)	2.79 (0.12)
O2(Cat) – S(SAM)	4.51	4.51 (0.08)	4.41 (0.05)	4.23 (0.08)	4.58 (0.08)	4.59 (0.09)
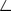 O2(Cat)-C(SAM)-S(SAM)	170	154 (8)	170 (2)	130 (8)	171 (5)	168 (6)
O(Asp141) – O(water)	2.77	2.55 (0.04)	2.51 (0.01)	3.05 (0.06)	2.54 (0.03)	2.49 (0.02)
O(Glu199) – O(water)	3.81	4.05 (0.27)	3.88 (0.33)	2.63 (0.02)	3.27 (0.19)	3.49 (0.18)

a Standard deviation are shown in parentheses; all values are in Å and degrees. ^b^Experimental values from 3BWM. ^c^After removing one outlier from the set of data: 2.23 (0.07) ^d^After removing one outlier from the set of data: 3.40 (0.06).

**Figure 4:**
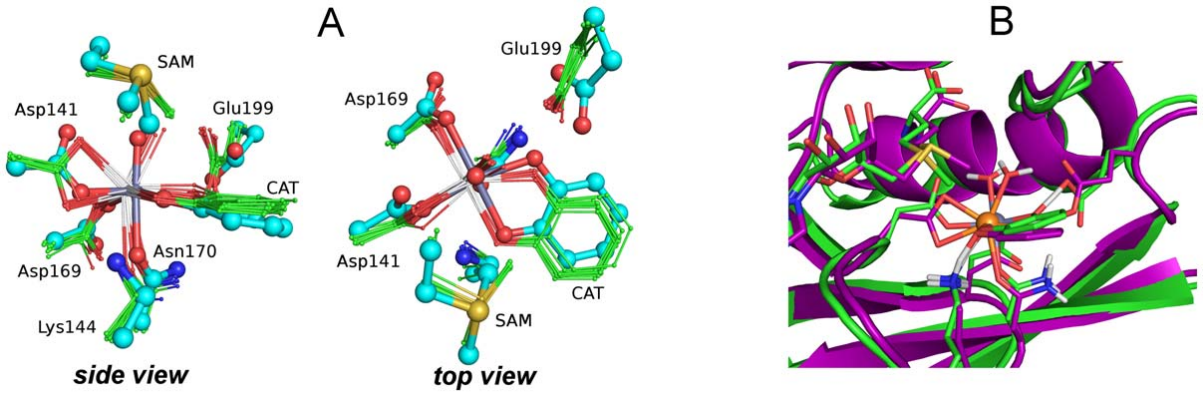
Comparison between Mg(II) and Ca(II) forms of COMT. A) Simulated active site for the Ca based COMT (thin sticks, small spheres) and the active site derived from the X-ray structure of Mg-COMT (bold sticks, large spheres). Hydrogen atoms, Met40, Lys46 and Asp205 have been omitted for clarity. B) The overlay of the representative snapshots from the QM/DMD simulations on Mg(II)-COMT (green structure, gray metal) and Ca(II)-COMT (purple structure, orange metal), showing the overall structural adjustment in Ca(II)-COMT.

When Mg(II) is replaced with Ca(II), a change is observed in the structure of the protein. Figure 4A shows the ensemble representation of the active site of COMT with Ca(II) in the active site, compared to the X-ray structure of the Mg(II) form of the protein. The difference in structure between the Mg-COMT and Ca-COMT is obvious, and also confirmed by the structural data included in Table 1. Firstly, all the metal – ligand bonds are longer in the Ca-COMT, as expected, given the larger size of the Ca(II) cation. As a result, the structure of the entire protein is perturbed and pushed outward and away from the metal. Secondly, new interactions are established in the active site. Ca(II) acquires a new ligand: both the oxygen atoms of the side chain of Asp141 now form direct contacts with Ca(II). The distances between the metal and the carboxyl oxygen atoms are 2.51 and 2.46 Å, whereas for the Mg-COMT, the Mg-O2_Asp141_ distance was at least 1.2 Å longer than the Mg-O1Asp141 distance, both in the experiment and in simulations. Thirdly, the water molecule coordinated to the metal establishes a hydrogen bond with Glu199, as both the oxygen atoms of the carboxyl group of Asp141 are engaged in bonding with Ca(II).

Interestingly, because of the different adopted coordination environment, Ca(II) sits deeper in the binding cavity (closer to Asp141) as compared to Mg(II) (Figure 4B). This, in turn, brings catechol closer to the SAM cofactor: the distance between the sulfur atom on SAM and the oxygen receiving the methyl group is significantly shorter (4.23 Å) when the cation is Ca(II) than for any other metal (4.41–4.59 Å). The proximity of the substrate and SAM does not necessarily imply enhanced reactivity, since it is also accompanied by the loss of collinearity of the sulfur atom, methyl, and hydroxyl group of catechol required for efficient methyl transfer. 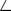
O(Cat)-C(SAM)-S(SAM) is 130°, as compared to 170° in the case of Mg-COMT.

We investigated the reliability of the QM/DMD method for structure prediction by using one of the structures predicted for Ca(II)-COMT as an input for an additional simulation of Mg(II)-COMT. QM/DMD was able to recover the native conformation of the Mg(II) protein, with one minor adjustment. Due to the fact that Ca(II) based structures have very long Metal – O1(Asn170) distances (ca. 2.63, compared to 2.14 Å), the QM optimization was unable to recover this short bond, as the side chain of Asn170 was diverted by other interactions. Thus, the simulation was formally trapped in a non–native conformation. A sufficiently long QM/DMD simulation would probably allow for the escape from the non-native state. However, by manually closing the gap between Mg(II) and Asn170 in the input structure, while leaving the rest of the geometry intact, we were able to recover the native conformation of the Mg(II) enzyme much more quickly.

Concerning the simulation involving Fe(II) and Fe(III) in the binding site of COMT, we found that the main features of the active site are consistent with the conformation assumed by the native enzyme containing Mg(II). The bond lengths reported in Table 1 are in agreement with the fact that the ionic radius of Fe is slightly smaller than that of Mg. Furthermore, except for the metal – O1(Asp141) and Metal – O2(Asn170), the ligand – metal bond lengths are smaller for Fe(III) than for Fe(II), in agreement with the greater electrophilicity of Fe(III). Convergence data for simulations on all proteins is presented in the (Figure S1, S2, S3, S4, S5). All obtained structures of metal-substituted COMT are available upon request.

### The methyl transfer step

All the structures resultants from the QM/DMD simulations were used as initial points (reactants) in a mechanistic study of the methyl transfer step. For every available structure of the reactant, the QM-DMD domain was extracted, unsatisfied valencies were capped with H atoms, and the atoms at the QM-DMD boundary were fixed as in the given structure of the protein. The structures of the TS to the methyl transfer step were then located and confirmed by the vibrational frequency analysis. The TSs contained the planar CH_3_ group in transit between the sulfur atom on SAM and the oxygen of catechol, in agreement with the S_N_2 reaction mechanism. The product adduct state where the methylated catechol was still coordinated to the metal was then also identified for each system (Scheme S2). In agreement with earlier reports, [19] we found that the H-bond between the catechol O atom and Lys144 is important in the reaction: as the methyl group transfers from S to O, Lys144 completely abstracts the proton from the substrate and becomes cationic.

Table 2 reports the averaged energies and significant structural parameters for the stationary points in the methyl transfer step catalyzed by the different metal forms of COMT, for the catechol substrate, and the inhibitor. The energy values are given relative to the energies of the reactant states. For Mg(II), the TS state is located 20.2±3.1 kcal/mol above the reactant state. This value in good agreement with the experimentally determined activation energy. The rate-determining step of the reaction is predicted to have a slightly positive energy. Structurally, the Mg(II) systems behaves as expected: The distance between the sulfur of SAM and the planar carbocation at the TS is 2.24 Å, and the distance between the carbocation and the O atom of catechol is 2.07 Å. The H-bond distance between the oxygen on catechol and the hydrogen that is being extracted by the side chain of Lys144 is progressively elongated during the course of reaction: from 1.24 Å in the reactant, to 1.53 Å in TS, and to 1.96 Å in the product. This goes together with the diversion of the electron density on O into the formation of the O-CH_3_ bond. This is true for all structures in the QM/DMD ensemble except for two that exhibit a different mechanism, where the H-transfer is already completed in the reactant state.

**Table 2:** Averaged over the QM/DMD ensembles energies (ZPE-corrected), and structural parameters (in Å) of the stationary points along the methyl transfer path, for the Mg(II), Ca(II), Fe(II), and Fe(III) forms of COMT, with the catechol substrate, and the Mg(II) form with the inhibitor.

	Energy		R(O–H_Lys144_)^a^			R(O–C)^b^	R(S–C)^c^
Structure	TS	Prod.	React.	TS	Prod.	TS	TS
**Mg(II) form**	20.2 (3.1)^d^	8.3 (5.8)	1.24 (0.20)	1.53 (0.18)	1.96 (0.37)	2.07 (0.03)	2.24 (0.01)
**Ca(II) form**	18.2 (3.3)	13.8 (3.4)	1.39 (0.03)	1.51 (0.02)	1.67 (0.03)	2.02 (0.02)	2.23 (0.06)
**Fe(II) form**	20.6 (3.3)	8.8 (7.8)	1.22 (0.11)	1.44 (0.05)	1.72 (0.03)	2.25 (0.10)	2.42 (0.07)
**Fe(III) form**	27.0 (5.7)	22.2 (6.7)	1.59 (0.25)	1.68 (0.23)	1.94 (0.30)	1.98 (0.03)	2.45 (0.03)
**Mg(II) form with inhibitor**	28.4 (4.7)	26.2(6.7)	1.70 (0.20)	1.89 (0.22)	1.98 (0.19)	1.96 (0.02)	2.37 (0.03)

a Distance between the oxygen atom of catechol and the closest hydrogen atoms on the side chain of Lys144, (see Scheme S2). ^b^Distance between the oxygen atom of catechol and the carbon atom on the migrating methyl group. ^c^Distance between the sulfur atom of SAM and the carbon atom on the migrating methyl group. ^d^Standard deviations are reported in parantheses.

The activation barrier for the Ca(II) form of COMT is lower than that for the Mg(II) form, 18.2 kcal/mol in spite of the lost collinearity of the methyl donor S atom, methyl group C, and the acceptor O, which was described above. However, this reaction step is predicted to have significantly higher overall energy. This is a manifestation of a pronounced inhibiting effect of Ca(II). Another difference is observed in the H-bond between catechol and Lys144, which was short in Mg(II) -COMT (1.24 Å), and becomes longer upon replacement of Mg with Ca (1.39 Å).

For Fe(II) bound to COMT, the activation energy is 20.6 kcal/mol and the overall energy of this reaction step is slightly positive, as for Mg(II). The structure of the active site and its evolution in the course of the reaction are very similar to those found for the native Mg(II) form of the enzyme. This is in agreement with the very similar performance of the two COMT variants, with the Fe(II) form being slightly inferior in terms of the catalytic activity.

For Fe(III), the barrier is higher, 27.0 kcal/mol, and the reaction is unequivocally up-hill (Table 2). The structural parameters that characterize the catechol oxygen environment during methyl transfer are found to be impacted significantly by the presence of Fe(III) in the active site. The H-bond between Lys144 and O of catechol is significantly elongated (1.59 Å) in the reactants, basically indicating that H is transferred to Lys144 already in the reactant state. This is a manifestation of the reduced basicity of the O of catechol bound to more electrophilic Fe(III), which undoubtedly prevents it from accepting the methyl group in the reaction. TS for Fe(III) COMT occurs very late, as compared to TSs characteristic of Mg(II), Ca(II) and Fe(II). This finding is in agreement with the Hammond–Leffler postulate and the relative energy of reactants and products.

When the substrate catechol is replaced with 3,5-dinitrocatechol, a typical inhibitor, in the active site of the Mg(II) form of COMT, the activation energy is significantly larger than in all other cases except for Fe(III), 28.4 kcal/mol. The reaction is also very endothemic. The H-bond distance between Lys144 and O of the inhibitor is largely elongated in the reactants, and the TS happens late. These observations are in agreement with a stabilization of the ionized catechol-COMT complex provided by the nitro groups.^16^ Thus, the inhibiting effect of 3,5-dinitro catechol is fully confirmed.

As the methyl transfer step is known to be rate limiting in the catalytic cycle of COMT, the relative activation energies are expected to provide useful insight in the understanding of the dependency of the enzyme's activity on the nature of the metal cofactor. From our results it follows that Mg(II) indeed gives the most active enzyme with an activation energy of 20.2 kcal/mol. In the case of Ca(II), the barrier is predicted to be slightly lower than in the case of Mg(II), but the reaction in the Ca(II) variant of COMT is significantly up-hill, suggesting inhibition. The electronic properties of Ca(II) are very similar to those of Mg(II). The reason for the observed difference in activity is due to large-scale protein adjustment and repacking effect, due to the larger size of the bound cation. In particular, it leads to misalignment of the methyl-donor and the acceptor, and various H-bonds stabilizing the product in the active site. QM/DMD was capable of capturing this effect. When the native Mg is substituted by Fe(II), the activation energy increases by ca 0.4 kcal/mol and such a value is compatible with the slightly lower activity experimentally observed for Fe(II)-COMT. On the other hand, the Fe(III) system has the computed activation energy of 27.0 kcal/mol and the reaction is strongly up-hill, in a full agreement with the experimentally determined inactivity of this system. The inhibiting effect of Fe(III) is thus solely due to the electronic properties of the metal. Coordination to the very electrophilic cation leads to the reduction in the basicity of O of catechol and reactants stabilization. Slight tightening of the entire coordination sphere of Fe(III) is also observed. Importantly, our QM/DMD simulations were also capable of comprehensively addressing this effect.

As a side note, for comparison, Table S1 contains the analogous data (energies and structures of the reactants, TS, and products) computed using a more standard cluster-based approach. Specifically, the active site of the enzyme was extracted from the available X-ray structure of the Mg(II) form, Mg(II) was replaced with other metals of interest, the points of attachment of the active site to the rest of the protein were fixed, and the system was optimized to the reactants, TS, and products, again confirmed with frequency analysis. In this way, the protein was not allowed to adjust to the changed size and charge of the cation. The activation energies obtained in this way disagree with the experimental data. Additional activation energies computed with different functionals and embedding schemes are reported in Table S2.

## Conclusions

We investigated the impact of different metal cations, Mg(II), Ca(II), Fe(II) and Fe(III), on the activity of the COMT enzyme. Our focus was the methyl transfer step between the SAM cofactor and the catecholic substrate, as it is known to be rate limiting in the catalytic cycle of COMT. Equilibrated ensembles of structures of the different metal protein variants were obtained via extensive sampling, achieved within our QM/DMD method, which in addition does not rely on any parameterization for the metals and describes them exclusively quantum mechanically. Structures of different metal variants differ in the architecture of the active site. The Mg(II) form of COMT obtained in simulations agrees very well with the available X-ray structure. Ca(II) adopts a different coordination: it captures the second O atom of the Asp141 side chain into its immediate coordination environment. As a result, the H-bonding arrangement in the active site changes. The overall coordination of Ca(II) is looser, i.e. not as organized and rigid, as in the Mg(II) enzyme. The substrate, the methyl group-to-transfer, and the methyl-donor S atom of the cofactor SAM, lose collinearity in the Ca(II) enzyme, harming the rate of methyl transfer. Fe(II) COMT is very similar to Mg(II) COMT. Finally, the Fe(III) form of the protein exhibits the overall tighter coordination of all ligands to the more electrophilic metal, including the substrate whose basicity is thereby compromised. All structures are available upon request.

The QM/DMD ensembles served as input for the mechanistic investigation. The rate-determining step of enzymatic catechol methylation was soundly described for all metal variants and the results are in the full accord with the available experimental data. In particular, we were able to demonstrate that the Mg(II) form of COMT is the most catalytically active. The inhibiting effect of 3,5-dinitrocatechol on the native Mg(II) COMT is also confirmed. For Fe(II), the activation energy increases by ca. 0.4 kcal/mol, which agrees with the fact that the Fe(II) substituted COMT is slightly less active. On the other hand, Fe(III) was found to inactivate the enzyme due to a preferential stabilization of the ionized catechol-COMT complex (reactant). Enzyme deactivation in this case is a purely electronic effect of the metal. In the case of Ca(II), the energy of the rate-determining step of the reaction increased dramatically, as a result of protein structural adjustment and repacking, which is a large-scale effect.

Therefore, we conclude that the dependence of the activity of COMT on the nature of the bound metal has different origins for different metals: it can be electronic (for Fe(II), and Fe(III)), or it can be large-scale structural (for Ca(II)). These effects are induced by the bound metal. The size of the metal impacts the quality of the alignment of the reacting molecules. Larger Ca(II) pushes the reactants apart, and this leads to misalignment of the methyl-donor and the acceptor, and of H-bonds stabilizing the product bound to the active site. This, in turn, leads to the increase of the reaction energy. The partial charge on the metal also matters, as it impacts the basicity of the catechol oxygen atoms, thereby changing the barrier at the electronic level.

Finally, we also would like to remark on the performance of QM/DMD, which again proved itself as a method capable of a reliable description of metalloproteins at a variety of scales, from large (1–10 Å, and beyond), to small (electronic level). It is suitable for modeling both native, and non-native, metalloproteins for which the experimental structures might be unavailable.

## Supporting Information

Figure S1 **Convergence data for the QM/DMD simulation of Mg(II) form of COMT: the RMSD of the protein backbone, the all-atom RMSD of the QM-DMD region, and QM energies as a function of iteration number.**
(TIF)

Figure S2 **Convergence data for the QM/DMD simulation of Ca(II) form of COMT: the RMSD of the protein backbone, the all-atom RMSD of the QM-DMD region, and QM energies as a function of iteration number.**
(TIF)

Figure S3 **Convergence data for the QM/DMD simulation of Fe(II) form of COMT: the RMSD of the protein backbone, the all-atom RMSD of the QM-DMD region, and QM energies as a function of iteration number.**
(TIF)

Figure S4 **Convergence data for the QM/DMD simulation of Fe(III) form of COMT: the RMSD of the protein backbone, the all-atom RMSD of the QM-DMD region, and QM energies as a function of iteration number.**
(TIF)

Figure S5 **Convergence data for the QM/DMD simulation of Mg(II) form of COMT: with the inhibitor the RMSD of the protein backbone, the all-atom RMSD of the QM-DMD region, and QM energies as a function of iteration number.**
(TIF)

Table S1 **Energies (ZPE-corrected), and structural parameters (in Å) of the stationary points along the methyl transfer path, for the Mg(II), Ca(II), Fe(II), and Fe(III) forms of COMT, with the catechol substrate, and the Mg(II) form with the inihibitor.** The input structures come directly from the X-ray structure, with the metal being replaced, an no protein repacking or backbone motion allowed.
(DOCX)

Table S2 **Averaged over the QM/DMD ensembles energies (ZPE-corrected of the stationary points along the methyl transfer path, for the Mg(II), Ca(II), Fe(II), and Fe(III) forms of COMT, with the catechol substrate, and the Mg(II) form with the inhibitor computed with B3LYP (with the active site embedded into the charge distribution generated by the rest of the protein) and TPSSh (active site solvated with Conductor-like Screening Model (COSMO) continuum solvation model (ε = 20)).**
(DOCX)

Scheme S1 **Methylation of catechol substrates catalyzed by COMT.**
(TIF)

Scheme S2 **Simplified representation of the stationary points located in the mechanistic study of the methyl transfer step.**
(TIF)
