# Supplementary material for: How Metal Substitution Affects the Enzymatic Activity of Catechol-O-Methyltransferase
Source: PLoS One. 2012 Oct 8;7(10):e47172. doi: 10.1371/journal.pone.0047172 (PMC3466255; doi:10.1371/journal.pone.0047172)
Supplement: Table S1 — Energies (ZPE-corrected), and structural parameters (in Å) of the stationary points along the methyl transfer path, for the Mg(II), Ca(II), Fe(II), and Fe(III) forms of COMT, with the catechol substrate, and the Mg(II) form with the inihibitor. The input structures come directly from the X-ray structure, with the metal being replaced, an no protein repacking or backbone motion allowed. (DOCX) [file pone.0047172.s006.docx]

|  | **Energy** | | **R(O–H_Lys144_)^a^** | | | **R(O–C)^b^** | **R(S–C)^c^** |
| --- | --- | --- | --- | --- | --- | --- | --- |
| **Structure** | **TS** | **Prod.** | **React.** | **TS** | **Prod.** | **TS** | **TS** |
| **Mg(II) form** | 20.4 | 9.3 | 1.17 | 1.51 | 1.70 | 2.07 | 2.24 |
| **Ca(II) form** | 13.2 | -1.7 | 1.13 | 1.51 | 1.68 | 2.07 | 2.24 |
| **Fe(II) form** | 16.5 | 6.5 | 1.40 | 1.56 | 1.76 | 2.10 | 2.29 |
| **Fe(III) form** | 16.6 | 19.1 | 1.56 | 1.68 | 1.75 | 1.94 | 2.44 |
| **Mg(II) form**  **with inhibitor** | 23.7 | 22.7 | 1.64 | 1.81 | 1.91 | 1.96 | 2.36 |

^a^Distance between the oxygen atom of catechol and the closest hydrogen atoms on the side chain of Lys144, (see Scheme 2). ^b^Distance between the oxygen atom of catechol and the carbon atom on the migrating methyl group. ^c^Distance between the sulfur atom of SAM and the carbon atom on the migrating methyl group.
