# Supplementary material for: How Metal Substitution Affects the Enzymatic Activity of Catechol-O-Methyltransferase
Source: PLoS One. 2012 Oct 8;7(10):e47172. doi: 10.1371/journal.pone.0047172 (PMC3466255; doi:10.1371/journal.pone.0047172)
Supplement: Table S2 — Averaged over the QM/DMD ensembles energies (ZPE-corrected of the stationary points along the methyl transfer path, for the Mg(II), Ca(II), Fe(II), and Fe(III) forms of COMT, with the catechol substrate, and the Mg(II) form with the inhibitor computed with B3LYP (with the active site embedded into the charge distribution generated by the rest of the protein) and TPSSh (active site solvated with Conductor-like Screening Model (COSMO) continuum solvation model (ε = 20)). (DOCX) [file pone.0047172.s007.docx]

|  | **B3LYP** | | **TPSSh** | |
| --- | --- | --- | --- | --- |
| **Structure** | **TS** | **Prod.** | **TS** | **Prod.** |
| **Mg(II) form** | 17.1 (2.5) | 6.9 (5.9) | 14.2 (1.2) | -4.1 (2.4) |
| **Ca(II) form** | 18.6 (3.1) | 14.9 (3.0) | 16.3 (1.8) | 1.2 (2.2) |
| **Fe(II) form** | 18.1 (3.0) | 9.1 (8.6) | 16.6 (1.8) | -3.3 (2.8) |
| **Fe(III) form** | 25.3 (4.7) | 23.1 (7.5) | 22.9 (2.8) | 9.8 (3.5) |
| **Mg(II) form**  **with inhibitor** | 25.0 (4.3) | 24.8(6.1) | 19.9 (1.5) | 9.9 (1.9) |
