# Supplementary figures and images for: How Metal Substitution Affects the Enzymatic Activity of Catechol-O-Methyltransferase
Source: PLoS One. 2012 Oct 8;7(10):e47172. doi: 10.1371/journal.pone.0047172 (PMC3466255; doi:10.1371/journal.pone.0047172)

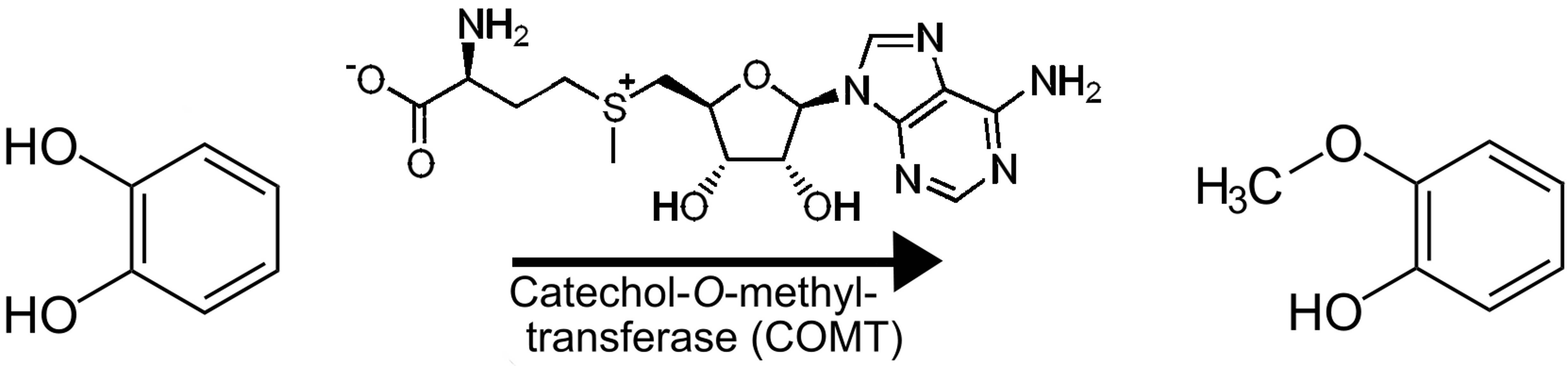

Supplement: Scheme S1 — Methylation of catechol substrates catalyzed by COMT. (TIF) [file pone.0047172.s008.tif]
